# Movable plates with g-C_3_N_4_/TiO_2_ as a compound system for a greener urban parking lot environment

**DOI:** 10.1371/journal.pone.0231286

**Published:** 2020-04-10

**Authors:** Chao Zeng, Mei Deng, Hao Qiao, Boming Tang, Yongjie Ding, Xuejuan Cao

**Affiliations:** 1 School of Traffic and Transportation, Chongqing Jiaotong University, Chongqing, China; 2 Chongqing Planning and Design Institute, Chongqing, China; 3 School of Civil Engineering, Chongqing Jiaotong University, Chongqing, China; 4 School of Architecture and Urban Planning, Chongqing University, Chongqing, China; 5 School of Material Science and Engineering, Chongqing Jiaotong University, Chongqing, China; University of California Santa Barbara, California, USA, INDIA

## Abstract

The application of photocatalyst in pavements has received comprehensive attention in recent years due to its ability to decontaminate nitrogen oxides (NOx). However, it’s remarkable that NOx also accumulated extensively in parking lots. The purpose of this study is to develop a movable photocatalytic plate (remarked photocatalytic KT plates) coupled with high activity to purify NOx. Firstly, the type of photocatalytic KT plates was determined according to NO removal experiment in laboratory. Then the plates were employed in the parking lots for removing NOx. One sample T-test, normality test and paired sample T-test methods for NOx concentration variation were conducted to determine the appropriate comparative means of dates under both dark and illuminated conditions. The difference of NO_X_ concentration between dark and illuminated conditions was obtained to evaluate the photocatalytic removal efficiency. The results indicated that NO removal efficiency in laboratory and parking lots were 51.31% and 9.2%, respectively.

## Introduction

Vehicle exhaust pollution is a key problem in the world environmental pollution. There are great amounts of nitrogen oxides (NOx), hydrocarbon (HC) and nitric oxide (CO) in the vehicle exhaust emissions [1–3]. Over the years, NOx always has a negative impact on human health, and numerous studies have proved this point. For example, Kim et al. [4] reported that NO_2_ may result in both asthma and bronchitis in children. The inhibition for vehicle exhaust concentration is a significant measure to improve environment. At present, there are three primary control measures about that: a) Administrative measures are effective strategies, such as the promulgation of mandatory automobile exhaust emission standards and regulations, improvement of traffic system, promotion of green travel and traffic restriction, etc. b) In-car cleaning measures include engine fuel design and use of new fuels. c) New materials with photocatalytic or absorption function can be applied to road surface and accessory facilities to purify automobile exhaust.

Among the three measures of above-mentioned, administrative and in-car clean measures primarily focus on automobile design and fuel development. Although the emission of automobile exhaust is controlled to a certain extent, the management and implementation are not easy to achieve in the long term. However, the application of photocatalytic materials on road pavements for removing automobile exhaust has a great development potential. Photocatalytic pavement technology is a cheap, easy-to-operate and environmentally friendly technology that semiconductor photocatalysts are added into road materials for forming coatings on road surfaces. When pavement is irradiated by light source, the automobile exhaust reacts with the active particles of photocatalyst, and secondary products attach to the pavement surface. The products are washed away by rain or are absorbed by plants, eventually, which resulted in a low automobile exhaust concentration in the air.

Recently, Titania-based (TiO_2_-) and graphitic carbon nitride-based (g-C_3_N_4_- and rGO-) nanocomposite are two typical semiconductor photocatalysts in the pollutant’s degradation. The application of g-C_3_N_4_- and rGO- compound in the organic dye degradation was explored by Paul et al [5–7]. Photocatalyst were added into building materials for NOx removal. The application of photocatalysts in combination with concrete materials have been become a focus of attention in photocatalytic field [8–11]. TiO_2_ was coated on the concrete blocks surface, and the specimens’ ability for reducing NOx convention was studied by Beelden et al [12]. Poon et al. [13] used TiO_2_ as an aggregate and mixed into several different concrete surface. From these studies, photocatalytic technology has a significant effect for removing NOx. Furthermore, the applications of TiO_2_ photocatalysts on limestone, bricks, cement and lime mortars were successful [14,15]. The supporting material with high porosity and roughness shows higher exposure of photocatalyst, thus NOx removal efficiency of materials is increased in different degradation mechanisms [16].

In addition, the use of photocatalyst on asphalt pavements also is an innovative and effective method of air pollution laxation [17]. In Italy, TiO_2_ was sprayed on existing asphalt pavements to form a thin surface layer for relieving NOx pollution. Cao et al [18]. applied Ce-TiO_2_ photocatalyst into asphalt by direct mixing, surface spraying and coating method, which found that the surface spraying method has the highest NO removal efficiency. Subsequently, Cao and Yang [19–21] et al. added g-C_3_N_4_/TiO_2_ composite into cationic emulsified asphalt and matrix asphalt to remove NO. It was found that the wear resistance of specimens accords with the road standard requirements, and the specimens still has good photocatalytic performance after wearing. However, the study shows that NOx removal efficiency was poor, which was strongly related to the properties of asphalt [22]. Black color and compactness problems have been considered as main concerns that result in a low pollutant removal efficiency of photocatalytic asphalt pavement.

Past researches mainly focused on the application of photocatalysts on road pavements. However, it is worth noting that NOx also accumulated seriously in parking lots that belong to semi-open environment. The semi-enclosed and non-ventilated environmental conditions of parking lots could cause an extensive increase of NOx and limit its dissipation when vehicles stop and start. Further, the interaction between NOx with exposed areas of building materials has a direct unfavorable impact on its functionality and aesthetic appearance. Accumulative dirt or NOx will worsen human health [23, 24]. To this aim, it is very necessary to reduce NOx pollution concentration and, consequently, to cut down the adverse impact on parking lots by using photocatalytic technology, which supports directing future research towards the use of photocatalytic materials in such environment.

### Objective

The objective of this study was to prepare highly active photocatalytic movable plates for removing NO and NOx under parking lots that are provided with semi-open environment conditions. Development and application of this new class of sustainable plates has the potential to degrade the convention for NO and NOx accumulated in parking lots. To achieve this objective, the photocatalytic removal efficiency of movable plates was tested and evaluated in the laboratory and in parking lots filed.

## Materials and experimental program

### Materials

Melamine and TiO_2_ were used to prepare g-C_3_N_4_/TiO_2_ composite. Foam plates (named as KT plates) was used as loading materials. Waterborne epoxy (BH-623) and curing agent (BH-519) were supplied by Dongguan Guangtong chemical products Co., Ltd (Guangdong, China) to product a mixed waterborne epoxy coating. Furthermore, Melamine (C_3_H_6_N_6_) were purchased from Aladdin Reagent Co., Ltd (Shanghai, China). TiO_2_ (anatase) was supplied by Hong-de Nanomaterials Co., Ltd (Nanjing, China). Deionized water was utilized in whole process of experiments.

### Preparation of g-C_3_N_4_/TiO_2_ composite photocatalyst

**Fig 1:**
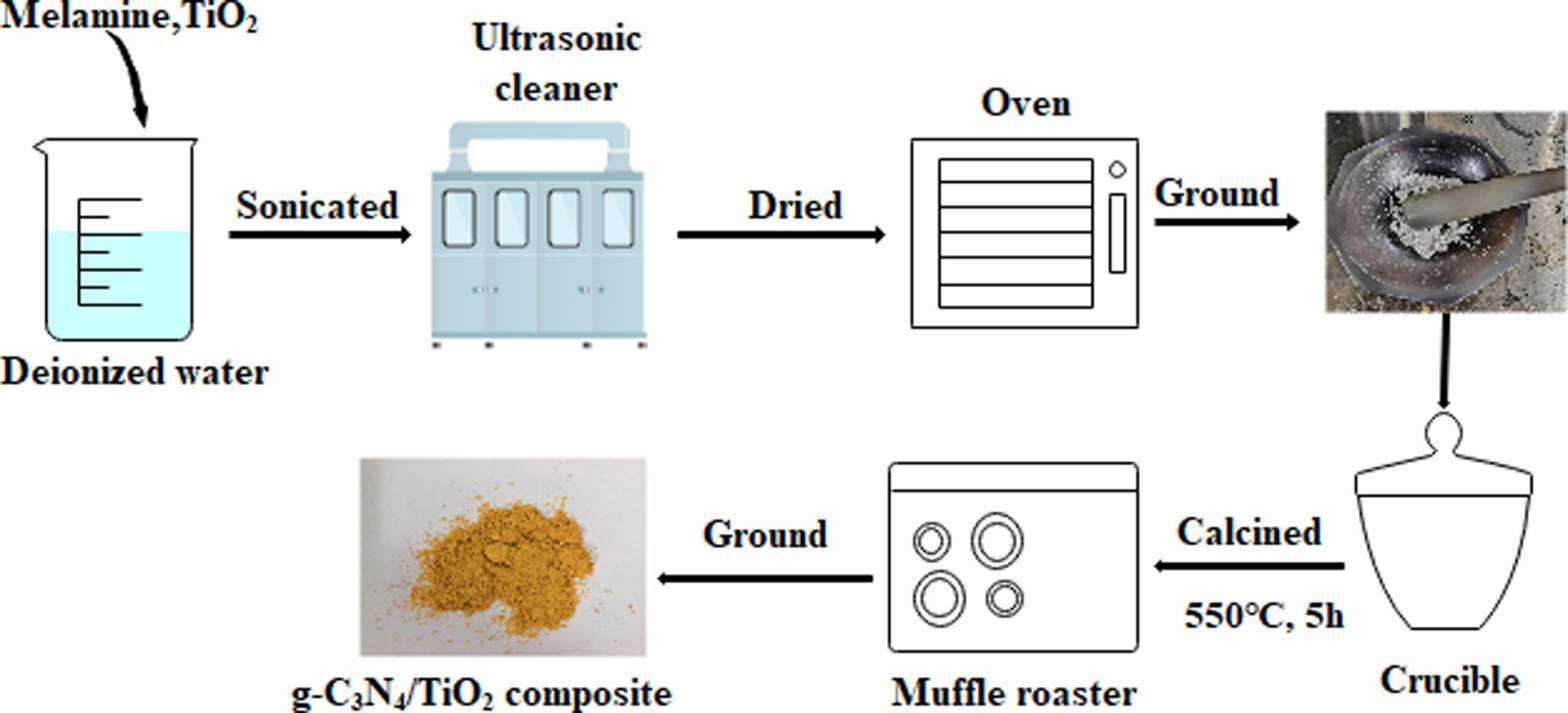
The preparation process of g-C_3_N_4_/TiO_2_ composite.

Both Melamine and anatase nanometer TiO_2_ monomer materials of 4:1 by mass were mixed and then dispersed ultrasonically for 30 min, and further dried at 80°C. The siccative solid mixtures were usually comminuted into powder. After that the powder was located on muffle furnace with a heating rate of 10°C/min, and then calcined at 550°C for 5 h. The mixture was ground into powder when it was self-cooled to room temperature for follow-up tests use. The preparation process was showed in Fig 1.

### Photocatalytic KT plates preparation

In this study, KT plates with a size of 90 cm Х 70 cm were chosen for loading photocatalysts, and the amount of photocatalyst was 12 g/m^2^. In addition, direct mixing method and surface spraying method were used to develop photocatalytic movable plates. In the direct mixing method, g-C_3_N_4_/TiO_2_ photocatalyst was added into waterborne epoxy resin. In a temperature range that does not impact properties of the waterborne epoxy resin [17], the mixture was evenly stirred by a shearer for 1 h, and then it was applied on KT plates. The thickness of photocatalyst waterborne epoxy resin coating was designed as 3 mm.

g-C_3_N_4_/TiO_2_ powdery materials usually result in an uneven dispersion and easy agglomeration in the waterborne epoxy resin, moreover, conventional methods of stirring cannot guarantee a proper dispersion of that. Therefore, in surface spraying method, the nano g-C_3_N_4_/TiO_2_ powder first was dispersed in water by ultrasonic cleaner. Then the mixed waterborne epoxy coating (m-_BH-623_: m-_BH-519_ = 1) was sprayed about 3 mm on the surface of the KT plates. Finally, g-C_3_N_4_/TiO_2_ photocatalytic aqueous solution was sprayed on the surface of waterborne epoxy resin thin layer using a hand spray gun when it was in the semi-curing state.

**Fig 2:**
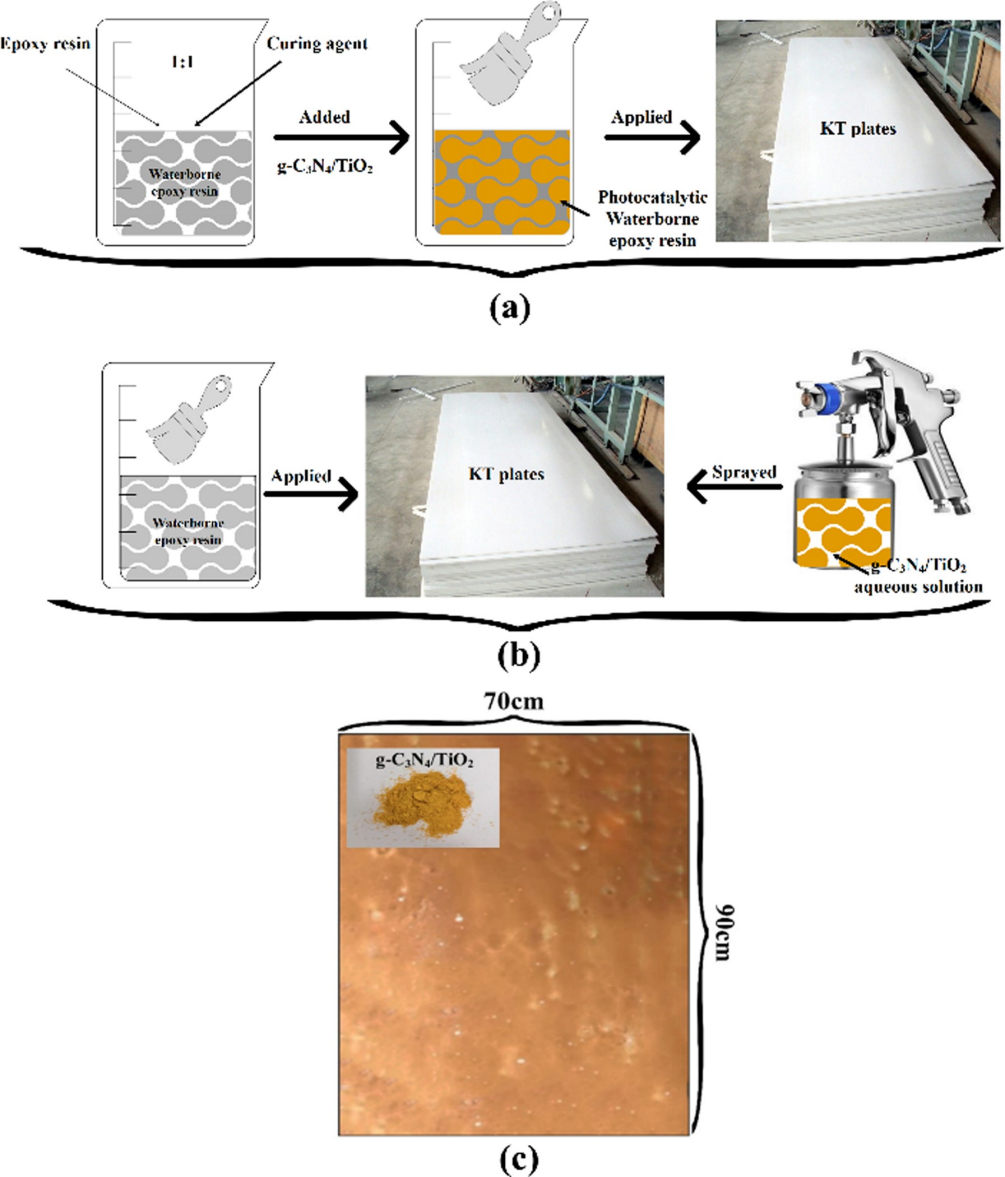
Preparation procedures of photocatalytic KT plates: (a) direct mixing method, (b) surface spraying method and (c) final specimens.

The as-prepared movable photocatalytic KT plates were kept at room temperature for drying. The preparation procedures were as shown in Fig 2A, 2B and 2C.

### Evaluation of photodegradation performance of NO

**Fig 3:**
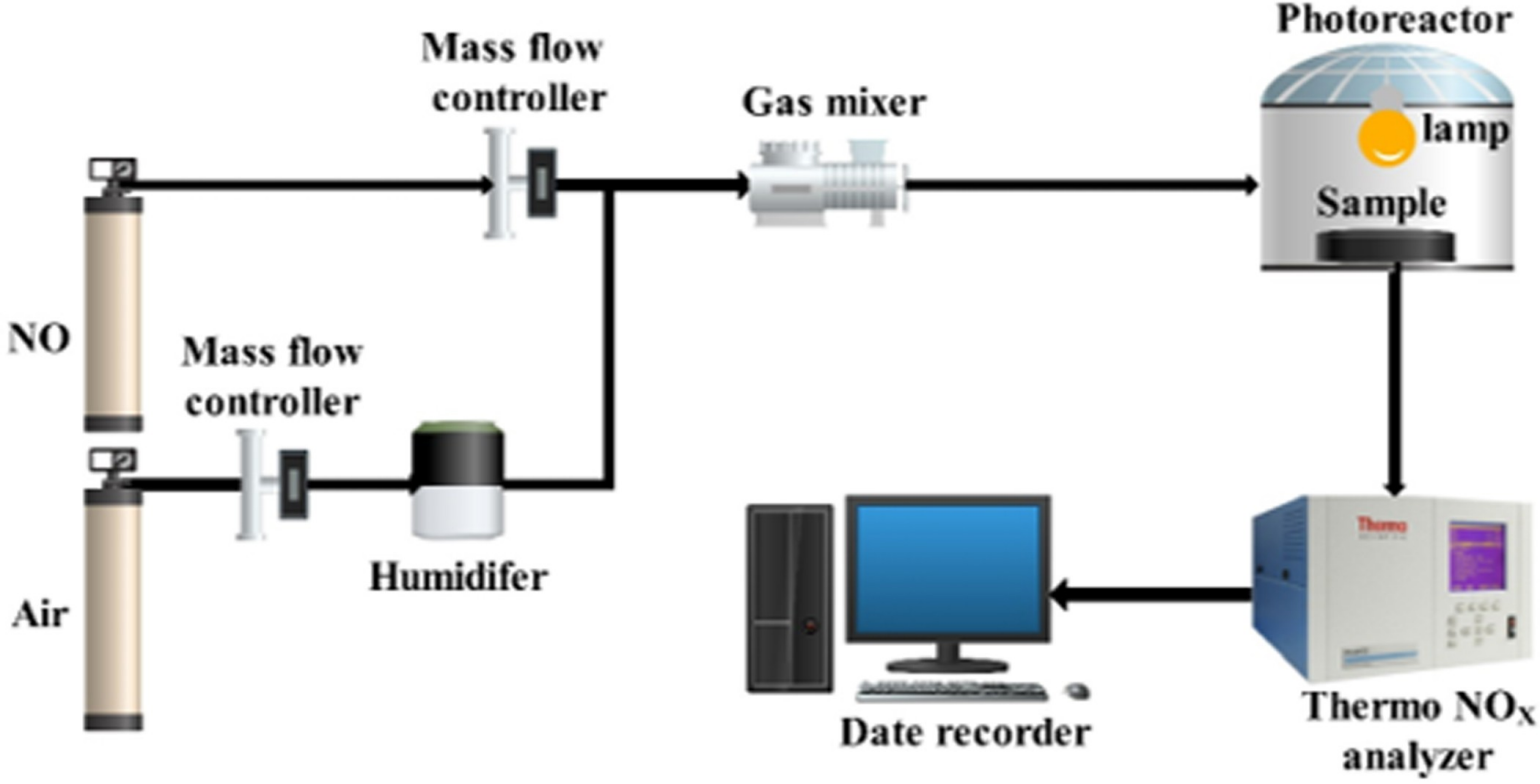
Schematic diagram of gas-phase photocatalytic degradation device.

In order to evaluate NO removal efficiency of photocatalytic materials, a gas phase photocatalytic degradation device was assembled as shown in Fig 3. The device consists of two high-pressure cylinders of NO and air, two mass flow controllers (FMA-A2000), a Bove Na B125 humidifier, a home-made gas mixer and a photoreactor (36cm×21cm×11cm), a Thermo-Scientific 42i NOx analyzer and a date recorder. Among them, the mass flow controllers and NOx analyzer were provided by Riga United Technology Co., Ltd. The main material quality of the photoreactor box was organic glass, and photoreactor cover was made of borosilicate glass with high light transmittance. Tin foil paper was adhered around the photoreactor to avoid the effect of external light source on test. In the process of removing NO, temperature and relative humidity were kept at 25°C and at 50%. NO and air were mixed as a pollutant source gas. The flow velocity of NO and air were controlled by two mass flow controllers, respectively. The pollutant gas of different concentration was simulated by adjusted mass flow controller. The pollutant source gas was inflowed the photoreactor. Then Thermo NOx analyzer recorded and inputted NO concentration in real time to the date recorder. The detailed degradation procedures were explained as follows.

To begin with, photocatalytic KT plates were placed in photoreactor, and flow velocity of air and NO were adjusted to 883 ml/min and 12 ml/min by mass flow controllers, respectively. Then, a metal halide lamp of 250 W (HALOPIN PRO|G9) was turned on for 60 and 120 minutes in laboratory and field experiment when the concentration was basically in equilibrium. Furthermore, when the reaction system was stable again, recorded the final NO concentration. Finally, NO removal efficiency can be defined as (1):

$$A=\frac{A_{0}-A_{1}}{A_{0}}X\;100\%$$ (1)

where *A*_*0*_ and *A*_*1*_ represent the initial and finally concentration of gas, respectively, and *A* is NO removal efficiency.

### Field experiment

**Fig 4:**
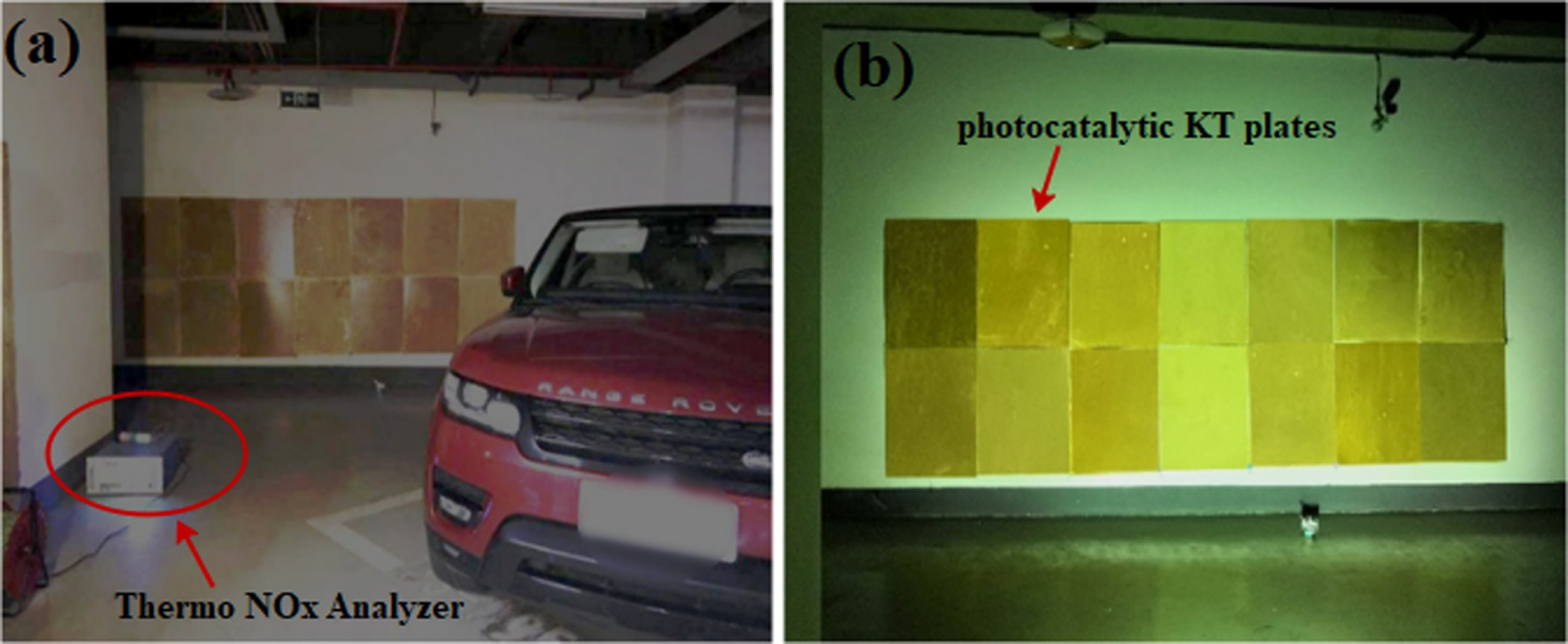
Field degradation experiments: (a) turn off the light, (b) turn on the light.

In this study, Star River Parking (it is a private land and the owner of the land gave permission to conduct the study on this site) located in Chongqing, China, was selected as test site for measuring NO and NOx removal efficiency. The parking area is 23,000 m^2^ with 445 parking spaces. The daily parking volume of Star River Parking from Monday to Friday is about 950 vehicles. Furthermore, a corner with poor air mobility in this parking lot was used as test point. Before photocatalytic KT plates application, the wall of the garage was cleared of any dust particles by sweeping. A prime coat of curing agent was applied prior to the photocatalytic KT plates pasting and then pasted 14 prepared specimens on that. 120 minutes was used as a node duo to various outdoor influencing factors for conducting two times test as shown in Fig 4A and 4B.

## Results and discussion

### Evaluation of NO removal efficiency in laboratory

**Fig 5:**
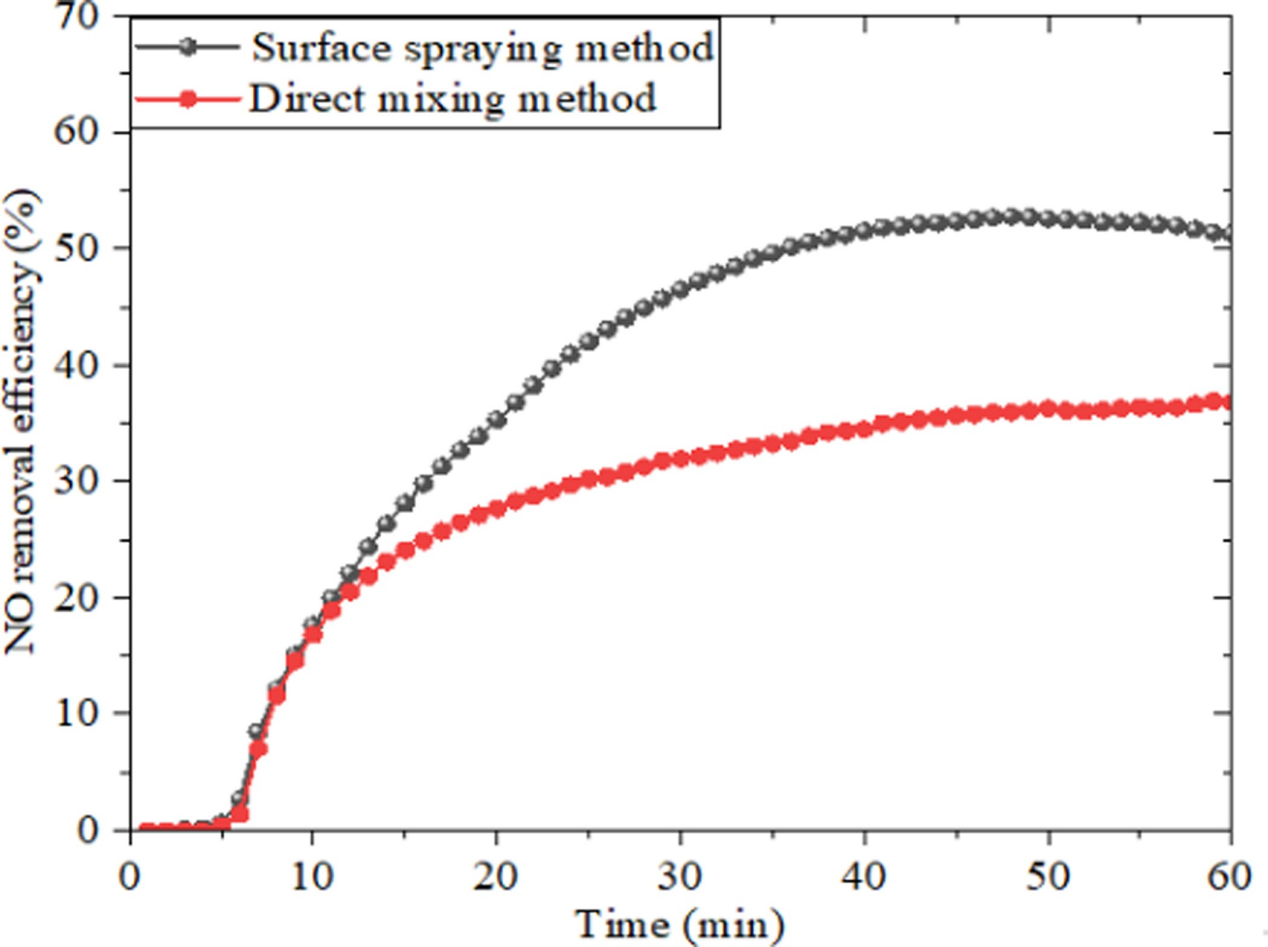
NO removal efficiency of direct mixing method and surface spraying method.

Fig 5 showed the trend of NO concentration variation of photocatalytic KT plates by using two preparation methods. It could be seen that NO removal efficiency ascended rapidly when the visible light source was turned on, and approached relative balance in about 40min. And apparently, the specimen with surface spreading method had significantly higher NO removal efficiency of 51.31%. The reason is that photocatalysts was distributed in the surface of waterborne epoxy resin film, which broken up agglomerate of g-C_3_N_4_/TiO_2_ materials and resulted in a large catalytic action area, thus, NO removal efficiency was improved. By contrast, NO removal efficiency of direct mixing method was 36.78%. The reason is that photocatalysts was enclosed into interior of waterborne epoxy resin system and further was easy to reunite. Based on the above analysis, surface spreading method was selected to prepare photocatalytic KT plates that were applied on parking lots.

### Concentration changes of NO and NOx

**Fig 6:**
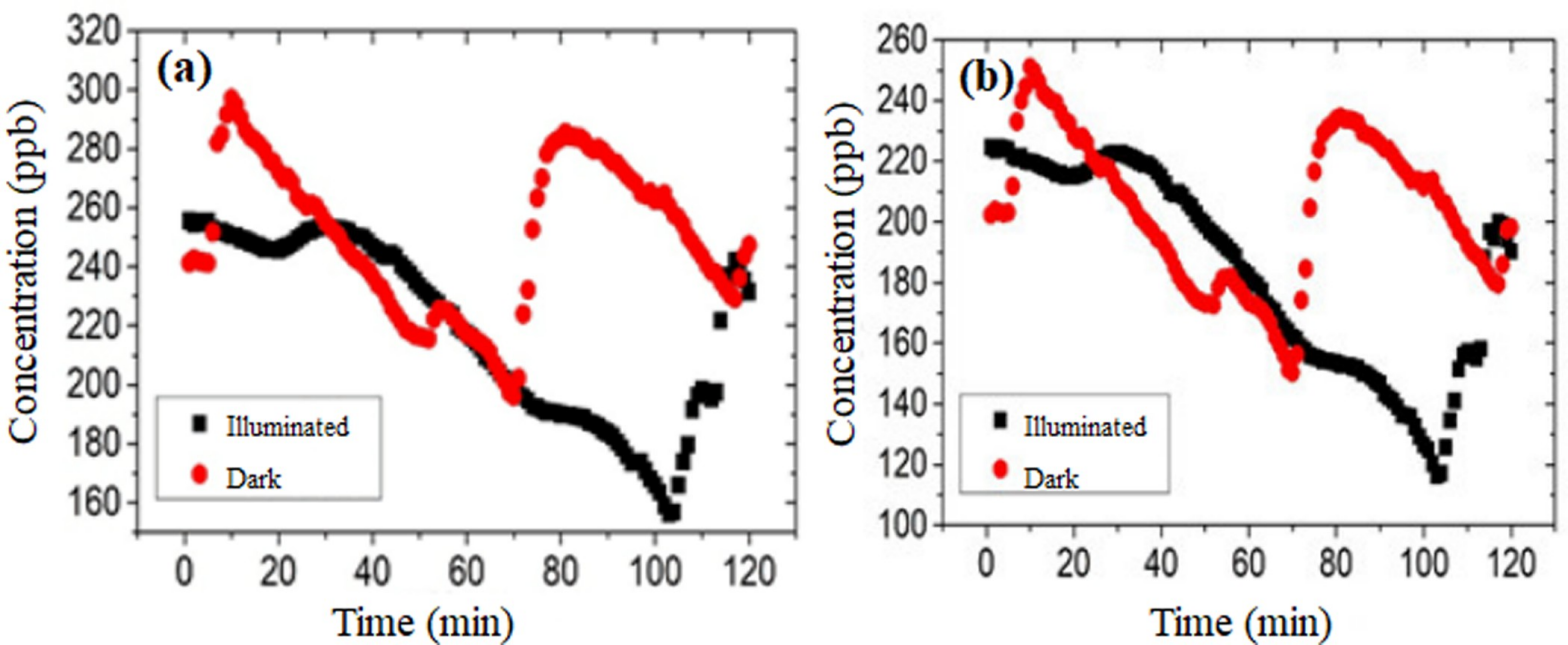
Concentration changes: (a) NO, (b) NOx.

The concentrations of NO and NOx in parking lots were measured under both illuminated and dark conditions. The results are showed in Fig 6A and 6B. It can be observed that NO and NOx concentration showed a similar change trend in the test process. Therefore, in this study, the concentration change of NO was only discussed because the conclusions still were suitable for NOx.

### Evaluation of NO removal efficiency in parking lots

As we all know, photocatalyst could produce an efficiency in illuminated condition. In this study, NO removal efficiency was evaluated by comparing with NO concentration under with and without light conditions. The distribution characteristics of the data was tested to determine the appropriate comparison method before comparing the two groups of data. Firstly, one sample T test was utilized to analyze two groups of concentration as shown in Table 1. It can be seen that the confidence interval of NO was 199.7 ppb-208 ppb and 177.8 ppb-189.8 ppb under both dark and illuminated conditions, respectively. The significance of two groups of data, however, were 0.00, which indicates that the concentration distribution may not conform to the normal distribution. Therefore, the concentration distribution was further analyzed.

**Table 1:** One sample T-test under dark and illuminated conditions.

Projects	Test value = 0					
	t	Degrees of freedom	Significance (2-tailed)	Mean difference	Difference 95% confidence interval	
					Lower limit	Upper limit
Dark condition	90.582	119	0.000	204.18139	199.7180	208.6448
Illuminated condition	60.648	119	0.000	183.85362	177.8510	189.8563

The normality test was carried out for two groups of data, and the results were showed in Table 2. It can be seen that the test significance of NO concentration distribution was more than 0.05 when the light was turned off, which suggested that the concentration distribution conformed to normal distribution. However, under illuminated condition, that of NO concentration distribution was 0.00, which indicates that data distribution was not followed to normal distribution.

**Table 2:** Normality test.

Projects	Kolmogorov-Smirnov test		
	Statistics	Degrees of freedom	Significance
Dark condition	0.079	120	0.063
Illuminated condition	0.157	120	0.000

**Fig 7:**
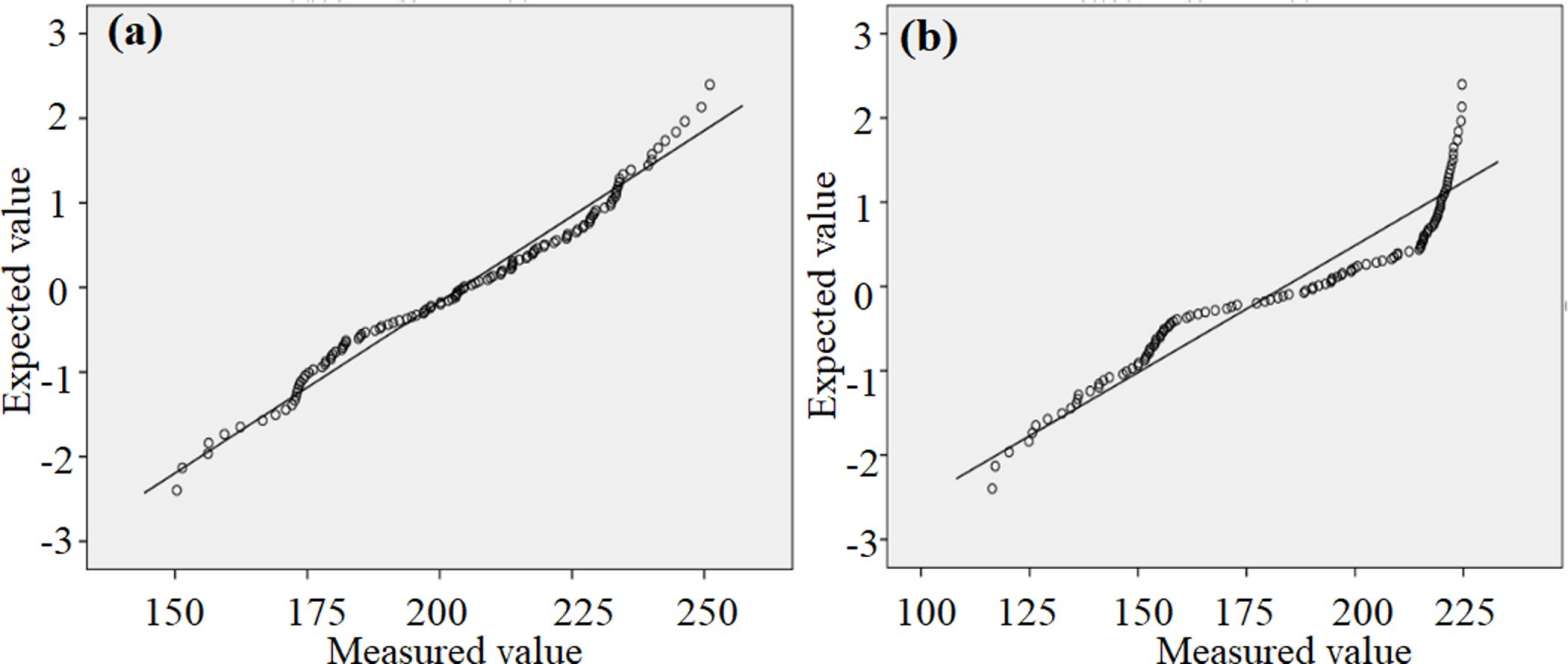
Q-Q diagram of concentration distribution: (a) dark condition, (b) illuminated condition.

**Fig 8:**
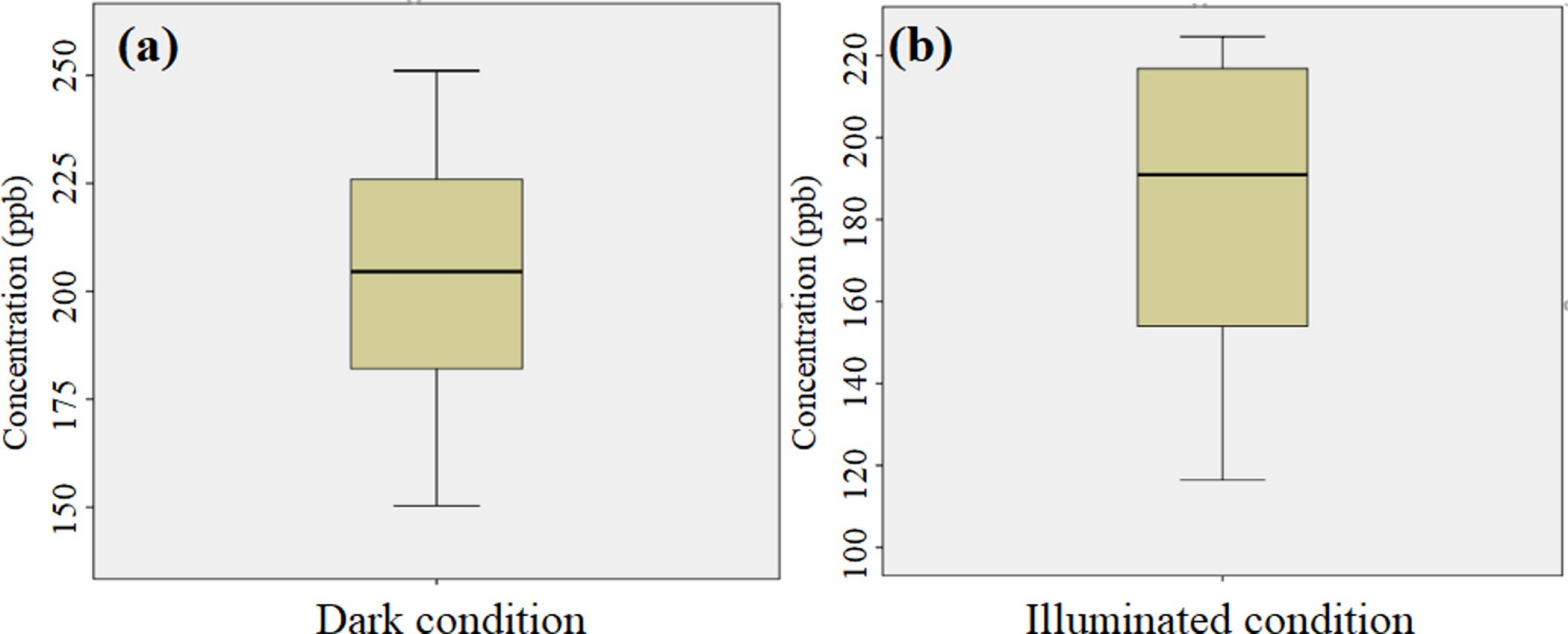
NO concentration distribution: (a) dark condition, (b) illuminated condition.

In order to further analyze the concentration distribution status, Q-Q and NO concentration distribution diagram were listed, respectively, as shown in Figs 7A and 7B, 8A and 8B. In Q-Q diagram, the concentration distribution was closer to the center line, which indicates that it was more inclined to the normal distribution. Meanwhile, it also can be found that NO concentration distribution was close to the center line when the light was turned off, and the average value was in the middle position, which indicates that NO concentration was not obviously interfered by external factors during test process. When the light was turned on, the average value was located above middle position, and the high concentration data deviated from the center line, which suggests that the changes of NO concentration were interrupted via photocatalysis.

From the above analysis, it was known that NO strength distribution accorded with normal distribution under the condition of turning off light source. Hence the paired sample t-test was further utilized to compare the varieties of gas concentration during both dark and illuminated states. The results were showed in Table 3, where the significance was 0.000, which infares that NO concentration changed significantly before and after turning on the lamp. The mean value of the difference between two states was 20.33 ppb, and the confidence interval of 95% was -27.69–12.96, which implies that NO concentration was reduced about 20.33 ppb.

**Table 3:** Paired sample test.

Projects		Paired difference					t	Degrees of freedom	Significance (2-tailed)
		Mean value	Standard deviation	Mean value of standard deviation	Confidence interval of 95%				
					Lower limit	Upper limit			
Paired 1	Turned on-off light	-20.32778	40.74522	3.71951	-27.69278	-12.96277	-5.465	119	0.000

In this study, NO concentration was about 220 ppb in the parking lot before the lamp was started. Based on the formula, NO removal efficiency was calculated of 9.2%.

## Conclusion and prospect

In this study, the NO removal efficiency of g-C_3_N_4_/TiO_2_ composite were investigated in laboratory and parking lot, respectively. First of all, photocatalysts loading methods on KT plates were determined by NO removal efficiency for laboratory. Then, NO concentration data of parking lots was inspected by using different sample test methods for analyzing the photocatalytic performance in parking lots. According to the above analysis, the following conclusions were obtained.

(1) The surface spreading method had a higher NO removal efficiency of 51.31%. The reason is that the agglomeration of g-C_3_N_4_/TiO_2_ composite was destroyed duo to the uniform distribution of photocatalyst on the surface of waterborne epoxy resin coating, which increased catalytic reaction area and resulted in an enhanced NO removal efficiency.

(2) NO concentration of both dark and illuminated conditions were not matched one sample T-test method. In normality test, NO concentration of dark conditions accorded with that, however, that of illuminated condition was opposite. The reason was that NO concentration was influenced by photocatalytic effect, which resulted in that NO concentration deviated from normal distribution. For paired sample T-test method, NO concentration was changed significantly before and after the lamp was turned on. The difference average value between two conditions was 20.3 ppb, which indicated that NO removal efficiency was 9.2% in parking lots.

(3) In the future research, more binders should be taken into account for the formation of surface spreading method, and the durability, wear resistance, photocatalytic properties of photocatalytic KT plates and its application cost will be evaluated.
